# Risk factors and mortality after elective and emergent laparatomies for oncological procedures in 899 patients in the intensive care unit: a retrospective observational cohort study

**DOI:** 10.1186/1754-9493-7-29

**Published:** 2013-09-05

**Authors:** Montserrat Mallol, Antoni Sabaté, Antonia Dalmau, Maylin Koo

**Affiliations:** 1 Department of Anaesthesia, Hospital Universitari de Bellvitge, IDIBELL, Universitat de Barcelona Health Campus, Barcelona, Spain; 2 Department of Anaesthesia, Acute Pain Clinic, Hospital Universitari de Bellvitge, IDIBELL, Universitat de Barcelona Health Campus, Barcelona, Spain; 3 Department of Anaesthesia, Surgical Critical Care Unit, Hospital Universitari de Bellvitge, IDIBELL, Universitat de Barcelona Health Campus, Barcelona, Spain; 4 Department of Anaesthesia and Reanimation, Hospital Universitari de Bellvitge, IDIBELL, Universitat de Barcelona Health Campus, Feixa Llarga s/n L’Hospitalet de Llobregat, Barcelona 08907, Spain

**Keywords:** Cancer, Abdominal surgery, Intensive care, Emergency, Postoperative complications, Mortality

## Abstract

**Background:**

Abdominal surgeries for cancer are associated with postoperative complications and mortality. A view of the success of anaesthetic, surgical and critical care can be gained by analyzing factors associated with mortality in patients admitted to intensive care units (ICUs). The objective of this study was to identify the postoperative mortality rate and the causes of perioperative death in high-risk patients after abdominal surgery for cancer. A secondary objective was to explore possible risk factors for death in scheduled and emergency surgeries, with a view to finding guidance on preventable risk factors.

**Methods:**

An observational study, in a 12-bed surgical ICU of a tertiary hospital. Patients admitted after abdominal surgery for cancer to the ICU for more than 24 hours’ care were included from January 1, 2008–December 31, 2009. Data were extracted from the minimum basic dataset. The main outcome considered was 90-day mortality.

**Results:**

Of 899 patients included, 80 (8.9%) died. Seven died within 48 hours of surgery, 18 died between 2 and 7 days, and 55 died after 7 days. Non-survivors were older and had more respiratory comorbidity, chronic liver disease, metastasis, and underwent more palliative procedures. 112 patients underwent emergency surgery; mortality in these patients for resection surgery was 32.5%; in the 787 patients who underwent scheduled surgery, mortality was 4.7% for resection procedures. The estimated odds ratios (95% confidence interval) of preoperative patient factors in emergency surgery confirmed a negative association between survival and older age 0.96 (0.91–1), the presence of respiratory comorbidity 0.14 (0.02–0.77) and metastasis 0.18 (0.05–0.6). After scheduled surgery, survival was negatively associated with age 0.93 (0.90–0.96) and chronic liver disease 0.40 (0.17–0.91). Analysis of complications after emergency surgery also indicated a negative association with sepsis 0.03 (0.003–0.32), respiratory events 0.043 (0.011–0.17) and cardiac events 0.11 (0.027–0.45); after scheduled surgery, respiratory 0.03 (0.01–0.08) and cardiac 0.11 (0.02–0.45) events, renal failure 0.02 (0.006–0.14) and neurological events 0.06 (0.007–0.5).

**Conclusions:**

As most deaths occurred after discharge from the ICU, postoperative sepsis, respiratory and cardiac events should be watched carefully on the ward.

## Abstract (es)

**Abstract:**

**Antecedentes**

La cirugía abdominal por cáncer se asocia a complicaciones postoperatorias y a mortalidad. El objetivo del presente estudio fue identificar la incidencia de mortalidad postoperatoria en pacientes intervenidos de cirugía abdominal por cáncer admitidos en una unidad de cuidados intensivos quirúrgicos (ICUs). Un objetivo secundario fue determinar los factores de riesgo mortalidad en base a la condición de la cirugía electiva o urgente.

**Método**

Estudio observacional durante el periodo Enero 1, 2008 a Diciembre 31, 2009 de todos los pacientes intervenidos de cirugía abdominal admitidos en una ICUs de 12 camas por un espacio superior a las 24 horas. Los datos fueron extraídos del conjunto mínimo de datos. La variable principal fue la mortalidad a los 90 días.

**Resultados**

Se incluyeron 899 pacientes, 80 (8.9%) fallecieron. Siete en las 48 horas de la cirugía, 18 entre el segundo y el séptimo día y 55 después. Los pacientes fallecidos eran de mayor edad, tenían asociadas patología respiratoria, afectación hepática, metástasis y los procedimientos quirúrgicos paliativos fueron más comunes. 112 pacientes fueron intervenidos de urgencia con una mortalidad para la cirugía resectiva del 32.5%; en los 787 pacientes electivos, la mortalidad fue del 4.7%. La Odds (intervalo de confianza 95%) de los factores preoperatorios en la cirugía urgente confirmó la asociación negativa entre la supervivencia y la edad 0.96 (0.91–1), la patología respiratoria 0.14 (0.02–0.77) y las metástasis 0.18 (0.05–0.6). En la cirugía electiva la supervivencia se asoció negativamente con la edad 0.93 (0.90–0.96) y con la patología hepática crónica 0.40 (0.17–0.91). Se observó una asociación negativa entre la supervivencia y la sepsis 0.03 (0.003–0.32), las complicaciones respiratorias 0.043 (0.011–0.17) y cardiacas 0.11 (0.027–0.45) en la cirugía urgente; mientras que en la cirugía electiva la asociación negativa con la supervivencia se obtuvo para las complicaciones respiratorias 0.03 (0.01–0.08), cardiacas 0.11 (0.02–0.45), el fracaso renal 0.02 (0.006–0.14) y las neurológicas 0.06 (0.007–0.5).

**Conclusiones**

La mayor parte de las muertes sucedieron después del alta de la ICU, y se asociaron a la sepsis y a las complicaciones respiratorias y cardiacas.

## Background

As surgical and anaesthetic techniques have increased in number and complexity, surgical outcome has remained closely related to the degree of deterioration of vital functions and is strongly influenced by the characteristics of the procedure [1]. Therefore, preventing major postoperative complications involving vital functions is central to improving results throughout the extended postoperative period, when early adverse events would be implicated [2].

Even though mortality is an unambiguous variable that can be calculated based on information from the minimum basic dataset and hospital discharge records [3], studies of mortality in oncological surgery have produced highly varied results and are difficult to apply in different clinical situations for many reasons. Oncological procedures account for many major abdominal surgeries, which are associated with substantially higher rates of postoperative complications and mortality [4]. Cancer itself leads to malnourishment and when exacerbated in patients who undergo preoperative chemotherapy, higher rates of postoperative surgical site infection have been observed [5]. Among the many additional factors affecting surgical outcomes, emergency status is one of the most important. Even if prompt access to operating facilities is possible, the rates of critical events and mortality are higher in emergency surgery [6]. This setting is therefore an invariable risk factor for mortality and can be a strong confounder that masks other risk factors when emergency and scheduled patients are studied together [7], accounting for some of the variability in results.

The provision of surgical intensive care units (ICUs) has been shown to improve outcomes [8], and it has been argued that because high morbidity has been reported for surgical patients on conventional wards [9], the number of critical care beds should be increased to facilitate safer management. Such arguments are based on culturally or medically context-based practices [10], however, and it is important to remember that about 12% of patients admitted to surgical ICUs die once they are transferred to a conventional ward [11].

A view of the success of this model of combining anaesthetic, surgical and critical care can be gained by analyzing factors associated with medium-term postoperative mortality in patients admitted to ICUs. An understanding of the causes of mortality would help to facilitates the redesign of management strategies for these high-risk surgical patients [12]. The main objective of this study was to identify the postoperative mortality rate and the causes of perioperative death in high-risk patients requiring more than 24 hours of surgical ICU care after abdominal surgery for cancer. A secondary objective was to explore possible risk factors for death in scheduled and emergency surgery patients, with a view to finding guidance on preventable risk factors susceptible to intervention.

## Methods

We extracted the minimum basic dataset for all adult abdominal surgery patients with cancer. The data for analysis encompassed all surgical processes and follow-up visits. Confidential patient information was protected, following national Spanish directives. This study was reviewed and approved by the clinical research ethics committee of Hospital Universitari de Bellvitge, which also reviewed the submitted manuscript to confirm compliance (IRB00005523).

The abdominal surgery patients had all been operated on at the same hospital and admitted to a 12-bed surgical ICU from January 1, 2008 through December 31, 2009. Patients who only stayed overnight in the ICU were excluded. Therefore, only first-admission patients who received critical care for more than 24 hour were included.

Scheduled surgery patients began preoperative anaesthesia evaluation in an outpatient visit; patients admitted through the emergency department, but scheduled for surgery, were also considered eligible as non-emergency patients. Emergency surgery patients were those admitted from the emergency department for urgent surgery (within 24 hours).

Abdominal surgery was classified according to the specific organ resected; surgery was considered palliative when resection was considered nonviable and a procedure to temporarily improve vital functions was performed. Anaesthetic management followed our hospital’s standard protocols, which included balanced general anaesthesia, perioperative warming of fluids and patients to maintain normothermia, routine antibiotic prophylaxis, and specific multimodal analgesia protocols (including patient-controlled opioid infusion and oral or intravenous non-steroidal anti-inflammatory drugs). Continuous epidural analgesia with local anaesthetics was used in patients undergoing gastric-oesophageal procedures or patients with advanced respiratory comorbidity, provided that no contraindications to central spinal blockade were on record.

Medical and surgical data for the preoperative, intraoperative and postoperative periods were collected. Respiratory, cardiac, renal, hepatic and neurologic comorbidities as well as respiratory, cardiac, renal and neurologic postoperative complications were defined according the descriptions in medical textbooks of reference [13,14]. The Charlson comorbidity index [15] and the surgical risk score (SRS) [16] were calculated from data recorded at admission We also calculated the American Society of Anesthesiology risk score.

The main outcome was in-hospital mortality or, when patients were discharged alive, 90-day mortality. This measure allowed us to include palliative surgeries in patients at very advanced stages of disease. Causes of death were classified as follows: sepsis when a systemic inflammatory syndrome was diagnosed and led to organ dysfunction and septic shock; respiratory complication when respiratory failure was present in spite of support ventilation; hemorrhagic shock when there was no response to blood replacement therapy, surgical treatment and/or haemostatic drugs; cardiac event (postoperative myocardial ischaemia, refractory heart failure or malignant arrhythmia); neurological event (severe postoperative stroke or refractory cerebral oedema); or cancer when progressive deterioration was directly related to the disease process. Cause-of-death classification was confirmed on the basis of information recorded in the medical history and considered both the root cause and the immediate cause of death. After initial assessment, all cases were re-evaluated by another researcher; in case of discrepancy, a third evaluator was consulted.

Other variables recorded were general patient characteristics, comorbidities, type of procedure, length of operation, transfusions, postoperative days in the ICU at first admission, surgical and medical complications, and total length of hospital stay.

### Statistical analysis

Data are presented as absolute number, percentage, mean and standard deviation, or median and range, as appropriate. The characteristics of survivors and non-survivors were compared; further comparisons for survivors and non-survivors in the emergency and scheduled subgroups were then made. We used the chi-square test or Fisher’s exact test for the comparisons, as appropriate; univariate analysis of variance was used for continuous homogeneous data. Statistical significance was set at *P* < 0.05. A maximum of ten variables found to be significantly associated with outcome in those comparisons were entered into multivariate logistic regression. In order to detect preoperative and postoperative predictors of mortality (negative survival), we considered each separately. Multivariate odds ratios and 95% confidence intervals (CI) were calculated. SPSS software version 15.0 (SPSS, Chicago, IL, USA) was used for all analyses.

## Results

During the period of study 899 patients who had undergone abdominal surgery for cancer were admitted to our ICU for stays longer than 24 hours. Eighty patients (8.9%) died; 39 had undergone emergency surgery and 41 scheduled surgery. Seven patients (4, emergency; 3, scheduled) died within 48 hours of surgery, 18 died between 2 and 7 days, and 55 died after 7 days (including 1 patient who died in-hospital after 115 days). Ten patients died during their first stay in the ICU (6 within 48 hours; 3 between 2 and 7 days and 1 after 7 days). The remaining 70 non-survivors died on the ward or during a subsequent stay in the ICU.

One hundred and twelve patients underwent emergency surgery; mortality in these patients was 32.5% for urgent resection surgery and 42% for urgent palliative surgery. In the 787 patients who underwent scheduled surgery, mortality was 4.7% for resection procedures and 12% for palliative procedures.

Overall, the rates of postoperative medical complications and reoperation were significantly higher in non-survivors. Respiratory causes led to the largest proportion of deaths (35%). Death was attributed to progression of cancer in 7 patients.

The 819 patients who survived and were discharged home were still alive after 90 days. Patient characteristics, comorbidity, surgical procedure and characteristics, and postoperative complications are summarised in Table 1. Non-survivors were older and had more respiratory comorbidity, chronic liver disease, and metastasis. Thus, these non-survivors had a larger total number of comorbid conditions and their SRSs were higher. The median length of hospital stay for survivors was 8 days (range, 5 to 51 days).

**Table 1:** Patient and procedure characteristics and causes of death

	**Non-survivors**	**Survivors**	***P *value**
	**(*n*** **= 80)**	**(*n*** **= 819)**	
Age, years	72.5 (65–81)	66 (57–73)	<0.001
Sex, male	68%	64%	0.6
Respiratory disease	31%	13%	<0.001
Heart disease	45%	44%	0.9
Chronic renal failure	10%	6%	0.12
Chronic liver disease	24%	11%	0.004
Neurological event	10%	9%	0.85
Diabetes	17.5%	18%	1
Metastasis	36%	22%	0.008
ASA classification (%)			
2	67	81	
3	30	17	
4	3	2	<0.001
Charlson index ≥ 3 (%)	51.25	47	0.483
SRS	7.9 ± 1.3	7.2 ± 1	<0.001
Urgent surgery	49%	9%	<0.001
Type of surgery			
Colorectal	43.75%	52.25%	
Gastric and oesophageal resection	15%	15.75%	
Liver and pancreatic resection	7.5%	18.5%	
Retroperitoneal resection	2.5%	1.5%	
Extended urologic resection	7.5%	3%	
Extended gynaecologic resection	2.5%	1%	
Palliative procedures	21.25%	8%	<0.001
Length of surgery, hours	3.1 ± 2	3.8 ± 2.1	0.005
Transfusion of blood products	5%	4.2%	0.7
ICU stay			
48 hours	66%	88.6%	
>48 ≤ days	5%	6.4%	
>4 days	29%	5%	<0.001
Postoperative complications			
Sepsis	30%	1.5%	<0.001
Respiratory	64%	4%	<0.001
Haemodynamic–cardiac	50%	4%	<0.001
Renal Failure	20%	1%	<0.001
Hepatic	10%	4%	0.23
Neurological	12.5%	0.5%	<0.001
Reoperation	24%	7%	<0.001
Cause of death			
Sepsis	22 (27.5%)		
Respiratory	28 (35%)		
Haemorrhagic shock	2 (2.5%)		
Cardiac	16 (20%)		
Neurological	5 (6.35%)		
Cancer	7 (8.75%)		

Data are expressed as median (full range of values), mean ± standard deviation, percentage, or absolute number (%). P value refers to analysis of variance and chi-square test when indicated. *ASA* American Society of Anesthesiology risk score, *SRS* Surgical Risk Score.

Univariate comparisons between surviving and non-surviving patients are shown according to emergency or scheduled surgical status in Table 2. Among both types of patient, non-survivors were older and had more respiratory comorbidity. Among emergency patients, non-survivors had significantly more extensive cancer (metastasis in 49%); consistent with this finding, significantly more palliative procedures were performed in these non-survivors. In contrast, among scheduled patients, non-survivors had more complex procedures and required more blood transfusions. Certain postoperative complications (sepsis, respiratory, cardiac and renal complications) differed between scheduled survivors and non-survivors. Although reoperation rates were relatively high in survivors, especially in emergency patients, differences between survivors and non-survivors were identified. Scheduled surgery patients who died had significantly more hepatic complications in all categories. Progression of cancer as the cause of death was higher in emergency patients.

**Table 2:** Patient and procedure characteristics and causes of death in emergency and elective surgery survivors and non-survivors

	**Emergency**		**Scheduled**		***P *value**
	**(*n*** **= 112)**		**(*n*** **= 787)**		
	**Non-survivors**	**Survivors**	**Non-survivors**	**Survivors**	
	**(*n*** **= 39)**	**(*n*** **= 73)**	**(*n*** **= 41)**	**(*n*** **= 746)**	
Age (years)	70 (64–71)	66 (54–74)	74 (69– 81)	66 (57–73)	<0.001
Sex (male)	61.5%	63%	73%	64%	0.34
Respiratory disease	31%	8%	32%	14%	0.005
Heart disease	41%	51%	49%	43%	0.52
Chronic renal failure	15%	6.8%	4.9%	5.4%	0.075
Chronic liver disease	17%	9.6%	7.3%	7.1%	0.262
Neurological event	10.2%	3%	9.7%	10.5%	0.242
Diabetes	15.4%	22%	19.5%	17%	0.735
Metastasis	49%	15%	24%	23%	0.85
ASA classification (%)					
2	67	84	67	81	
3	31	16	29	17	
4	3	0	2.5	2	0.064
Charlson Index ≥ 3 (%)	38.5	48	58.5	53.6	0.1
SRS	8.7 ± 1	8.9 ± 1	7 ± 1	7 ± 0.7	<0.001
Palliative surgery	28%	23%	15%	7%	0.01
Length of surgery, hours	2.54 ±2	3 ± 2	3.8 ± 2	4 ± 2	<0.001
Transfusion of blood products	5%	4.2%	12.3%	3.2%	0.003
Postoperative complications					
Sepsis	28%	4%	32%	2%	<0.001
Respiratory	61.5%	8%	66%	3.5%	<0.001
Haemodynamic-cardiac	51%	10%	49%	4%	<0.001
Renal Failure	15%	3%	24%	1%	<0.001
Hepatic	0%	5.5%	19.5%	4%	<0.001
Neurological	10%	1%	15%	0.5%	<0.001
Reoperation	23%	15%	25%	6%	<0.001
Cause of Mortality					
Sepsis	10 (25.6%)		12 (29%)		
Respiratory	13 (33.3%)		15 (36.6%)		
Haemorrhagic shock	—		2 (4.9%)		
Cardiac	8 (20.5%)		8 (19.5%)		
Neurological	3 (7.8%)		2 (4.9%)		
Cancer	5 (12.8%)		2 (4.9%)		

Data are expressed as median (range of values), mean ± standard deviation, or absolute number (%). P value refers to analysis of variance and chi-square test when indicated. *ASA* American Society of Anesthesiology risk score, *SRS* Surgical Risk Score.

The multivariate analysis of patient factors in emergency surgery confirmed a negative association between survival and older age, the presence of respiratory comorbidity and metastasis. After scheduled surgery, survival was negatively associated with age and chronic liver disease. Neither the Charlson index nor the SRS was associated with mortality in either group (Table 3). Multivariate analysis of postoperative complications confirmed the negative association between nearly all medical postoperative complications and survival in both scheduled and emergency surgery; however, emergency patients differed in that sepsis was a significant predictor of mortality (negative association with survival) but renal and neurological complications were not (Table 4).

**Table 3:** Multivariate analysis of causative preoperative factors associated with survival

	**Emergency**	***P *value**	**Scheduled**	***P *value**
	**(*n*** **= 112)**		**(*n*** **= 787)**	
Age	0.96 (0.91–1)	0.05	0.93 (0.90–0.96)	<0.001
Sex, male	0.84 (0.27–2.58)	0.76	0.69 (0.32–1.48)	0.34
Respiratory disease	0.14 (0.02–0.77)	0.02	0.55 (0.23–1.34)	0.19
Chronic liver disease	0.98 (0.24–3.91)	0.98	0.40 (0.17–0.91)	0.02
Metastasis	0.18 (0.05–0.6)	0.006	0.68 (0.30–1.58)	0.37
Charlson index	0.68 (0.14–3.2)	0.63	1.27 (0.45–3.57)	0.66
SRS	1.71 (0.98–2.98)	0.059	1.26 (0.81–1.96)	0.31
Length of surgery	1.14 (0.73–1.76)	0.56	0.93 (0.8–1.07)	0.58

Data are expressed as odds ratios (95% confidence intervals).

**Table 4:** Multivariate analysis of causative postoperative complications to survival

	**Emergency**	***P *value**	**Scheduled**	***P *value**
	**(*n*** **= 112)**		**(*n*** **= 787)**	
Complications				
Sepsis	0.03 (0.003–0.32)	0.004	0.28 (0.07–1.06)	0.062
Respiratory	0.043 (0.011–0.17)	<0.001	0.03 (0.01–0.08)	<0.001
Haemodynamic-cardiac	0.11 (0.027–0.45)	0.002	0.11 (0.03–0.35)	<0.001
Renal Failure	0.17 (0.012–1.1)	0.063	0.02 (0.006–0.14)	<0.001
Hepatic	1.37 (0.06–1.45)	0.99	0.35 (0.09–1.35)	0.13
Neurological	0.25 (0.01–6.44)	0.42	0.06 (0.007–0.5)	0.01
Reoperation	1.96 (0.15–26.2)	0.61	2.32 (0.53–10.11)	0.26

OR: odds ratio (95% confidence interval).

## Discussion

The 8.9% mortality in this series was similar to rates reported for other series of high-risk oncological surgery patients [17,18]. Most deaths occurred after postoperative day 7 and once the patients had been transferred from the ICU to a conventional ward. The high prevalence of comorbidity partially explains high incidences of both postoperative complications and mortality and is consistent with other studies [19]. Overall, our non-survivors were older, suffered from more chronic diseases and had more advanced cancer, reflected by the 36% prevalence of metastasis at the time of surgery.

Mortality was particularly high in emergency surgery patients, also consistent with the literature [6,20]. In this setting, the quality of the procedure may be undermined for various reasons and patient preparation may be less thorough [21]. When initially comparing survivors and non-survivors, we detected differences in relation to mortality, age, comorbidity, the SRS, emergency status and type of surgery. However, multiple regression analysis confirmed only age, preoperative respiratory disease and metastasis as predictors of mortality in emergency patients and only age and chronic liver disease in scheduled patients. Our findings support recent challenges regarding the association between overall comorbidity assessments, including risk indexes, and outcome and the call to consider more specific quantitative predictors of complications, such as inspiratory muscle endurance [22].

Major postoperative complications were clearly more common in non-survivors in both the emergency and scheduled subgroups. However, even though reoperation was more frequent in non-survivors, on multivariate analysis it was not confirmed as a predictor, in contrast with results from another series [23]. In that series, reoperation was related to sepsis, which is commonly caused by anastomotic leakage. In our series, multiple regression analysis confirmed that sepsis was associated with mortality only in emergency cases, possibly attributable to the relatively high rate of reoperation in emergency survivors. Avoiding non-steroidal anti-inflammatory drugs in colonic surgery has been suggested as a way to reduce anastomotic leakage [24], which is a main cause not only of reoperation but also of major medical complications. In a recent critical analysis of the evidence in support of increasing global blood flow, the rates of renal failure, respiratory failure, and wound infections were reduced when stipulated goals were obtained [25]. We feel that some of these interventions could help to protect patients from anastomotic leakage, reducing reoperation rates in our high-risk patients, and are candidates for testing in trials. However, we emphasize that we found that respiratory complications were more common among non-survivors overall and therefore agree with others that predicting postoperative pulmonary complications can facilitate the selection of those patients that could benefit from specific evaluation and preparation for surgery [26] as well as increased vigilance on the ward [12]. Preoperative physical therapy has been found useful in preventing respiratory complications [27].

Progression of disease was the cause of death in only 7 cases (8.8% of all patients who died), even though a high percentage of our patients had advanced cancer, 23.5% of the series had metastasis (with similar rates in survivors and non-survivors) and a palliative procedure was performed in 84 patients (10% of series). In addition, it is noteworthy that medical complications were common in patients who underwent palliative surgery. Death, whether during the first ICU stay or after first discharge from the ICU was most often attributed to respiratory or cardiac events or to sepsis.

Oncological procedures are mostly scheduled surgeries; therefore, figures from emergency cases are not large enough to evaluate such uncommon complications as hepatic and neurological issues. Also, mortality in emergency cases was very high, so that the differences between survivors and non-survivors were related to age, respiratory comorbidity and metastasis as in the series overall.

In one European multicentre study, 73% of patients who died had not been admitted to critical care at any stage after surgery [28]. Although in our series all patients were first managed in a surgical ICU, most complications that led to death occurred after ICU discharge. We therefore consider it to be highly advisable to implement some sort of programme for supervising high-risk patients more closely on wards, as suggested by Goldhill [12].

One limitation of our study was the retrospective nature of our intraoperative data collection, which potentially underestimated the influence of intraoperative events on postoperative outcome [29]. Another limitation was our use of the SRS, which does not incorporate physiological data to adjust the predictive value. Even given these limitations, however, we have gathered information that has helped us identify future strategies that may improve outcome.

## Conclusions

To summarize, as expected, postoperative mortality was high overall in our abdominal oncological surgery patients admitted to the ICU for more than 24 hours. Mortality was even higher after emergency procedures and in older patients with respiratory comorbidity and more advanced cancer, even though associated comorbidity in scheduled surgery has limitations to select high risk patients and it should be considered more specific quantitative predictors. Most deaths occurred after the patients had been initially discharged from the ICU. Reoperation was not associated with mortality. Sepsis and respiratory and cardiac events caused most deaths, and we conclude that these would be the complications to watch for very carefully inside the ICU and after discharge; we are therefore considering implementing a programme for supervising high-risk patients more closely on conventional wards.

## Abbreviations

CI: Confident interval; ICU: Surgical intensive care unit; SRS: Surgical risk score.

## Competing interests

No conflict of interests is declared by any of the authors.

This work was supported by the Research Foundation of the University Hospital of Bellvitge. (IDIBELL), which provided a data manager for data collection.

## Authors’ contributions

MM: interpretation of data, first draft of the article, final approval of the article. AS: Design, interpretation of data, first draft of the article, final approval of the article. AD: Design, interpretation of data, first draft of the article, final approval of the article. MK: Design, interpretation of data, final approval of the article. All authors read and approved the final manuscript.
